# English teachers’ perceptions of emergency remote teaching: Emotional attitudes, professional identity, and coping strategies

**DOI:** 10.3389/fpsyg.2022.1064963

**Published:** 2023-01-12

**Authors:** Fang Huang, Mingyan Zhao, Jiafu Qi, Ruyue Zhang

**Affiliations:** Foreign Languages Educational Research Center, School of Foreign Languages, Qingdao University, Qingdao, China

**Keywords:** emergency remote teaching, emotional attitudes, strategies, professional identity, experienced English teachers

## Abstract

While the technology integration has been widely acknowledged, insufficient effort has been made to understand English teachers’ perceptions of emergency remote teaching (ERT). Given English is the primary foreign language in China and English teachers’ perceptions and adoptions of ERT impact the attainment of teaching and learning goals, this study inquired into experienced university English teachers’ emotional attitudes, perceptions of the reconstructed professional identity, and their strategies to cope with difficulties when conducting ERT in the Chinese English teaching context. Semi-structured interviews were conducted both online and face-to-face with five experienced universities English teachers in China. The findings indicated a trajectory of teachers’ attitudes toward ERT, namely, their attitudes were switched from doubt and rejection to fondness and attachment. Experienced English teachers adopted various strategies to cope with the difficulties and challenges they have encountered, including seeking support from their peers, students, and family, as well as self-regulated learning to sustain continuing professional development. During ERT, they have reconstructed their teacher identities. The study enriched peoples’ understandings of English teachers’ perceptions of the ERT by contextualizing the study in the Chinese educational context. Results can provide empirical evidence for policymakers and teacher trainers to make informed decisions regarding technical support and teachers’ continuing professional development.

## 1. Introduction

With the pervasiveness of Information and Communication Technology (ICT) in education, technology-assisted language teaching and learning have gradually become a norm (Teo et al., 2018; Huang et al., 2019, 2020; Dong and Ishige, 2022). Recently, technology tools, such as laptops, mobile phones, projectors, and multimedia, have been widely used by language teachers in informal and formal teaching settings, and the integration has transformed teachers’ pedagogy and brought about benefits for teaching and learning (Kvavik, 2005; Herrington and Kervin, 2007; Baek et al., 2008; Shadiev et al., 2018; Tusmagambet, 2020). Its success highly depends on external factors (e.g., technical support, time; Ifenthaler and Schweinbenz, 2013; Siyam, 2019) and internal factors including language teachers’ attitudes toward technology use (Teo et al., 2018; Huang et al., 2019), their beliefs (e.g., Leem and Sung, 2019), self-efficacy (Ertmer, 1999; Huang et al., 2019), design-thinking in teaching and teacher identity (Tsai and Chai, 2012), so on and so forth. These factors (e.g., self-efficacy, social support) further influence teachers’ strategy choices, positive coping with stress (Linley and Joseph, 2004; Shen, 2009) and perceptions of teacher identities when teaching with technologies (Belland, 2009; Kim et al., 2013; Rauf and Suwanto, 2020).

When the COVID-19 pandemic broke out, English teaching has been shifted from traditional classroom-based teaching to online remote teaching, and scholars named this sudden shift as the emergency remote teaching (ERT; Hodges et al., 2020; Mohmmed et al., 2020; Huang F. et al., 2021). It is noticeable that ERT is not complementary to classroom teaching and instead, it is the only effective way for English teachers to sustain teaching from disruption caused by unexpected events (Hodges et al., 2020). Therefore, governments in diverse countries and regions issued policies to promote ERT and provide support for teachers. The unprecedented shift caused changes in teachers’ attitudes toward technology use (Galloway, 2017; Hong et al., 2020) as well as teaching strategies, and accordingly, reconstructed their identities (Yan and Wang, 2022). Scholars need to revisit these given the paucity of studies in the changing research context.

Previous studies in technology-assisted language teaching and learning mainly adopted quantitative method to examine teachers’ perceptions of using technology (e.g., perceived usefulness, ease of use, complexity), teacher-perceived self-efficacy and attitudes (e.g., Hsu, 2016; Teo et al., 2018; Huang et al., 2019; Huang F. et al., 2021), these studies led to peoples’ understanding of the general situation but cannot provide a rich understanding of teachers’ perceptions, given quantitative research (usually using the randomized approach to gather data) only provides specific facts but does not care about individuals’ thought processes when sharing an opinion or making a choice, nor it considers the meaning behind social phenomena. Comparatively, qualitative research concerns aspects of reality that cannot be quantified, it aims to produce in-depth and illustrative information so as to understand the various dimensions of the problem or phenomena under analysis (Maxwell, 2013; Queirós et al., 2017).

In the existing literature, few adopted qualitative methods to inquire into English teachers’ attitudes, professional identity, and pedagogical strategies regarding technology use (Lai and Jin, 2021), and even fewer were conducted in the ERT setting (Yan and Wang, 2022). Some studies introduced ERT of K-12 teachers (Trust and Whalen, 2020; Arcueno et al., 2021) and pre-service teachers (Karataş and Tuncer, 2020; Mohamad et al., 2020; Sumardi and Nugrahani, 2021), but failed to unpack teachers’ perception change, which is a dynamic process. Furthermore, existing qualitative studies did not place the foci on elderly and experienced teachers in university, who might perceive differently from their younger peers in terms of using technology in teaching. Teachers who are previously voluntary technology users (Chung et al., 2019) became mandatory or non-volitional users (Huang F. et al., 2021). In order to sustain teaching and learning in the emergency context, they have transformed pedagogy, innovated teaching and assessment, and picked up skills to use mobile tools and personal computers (PC). These will definitely exert influence on their ongoing teaching concept and practice in formal and informal settings. Furthermore, ERT entails mobile tools in teaching and learning, however, existing studies on mobile-assisted language learning have been mostly in the formal educational contexts (e.g., classroom trials or experiments of mobile applications), MALL in informal contexts is a less explored territory to date and researchers are keen to understand teachers’ use of mobile devices beyond the classroom (Viberg and Grönlund, 2013; White and Mills, 2014; Kukulska-Hulme et al., 2017; Lai and Zheng, 2019). Thus, this study aimed to bridge the gap in the literature by inquiring into experienced university language teachers’ attitudes toward ERT, their identities, and coping strategies during the pandemic. In doing so, this study will enrich the existing literature on mobile-assisted language teaching and learning and provide practical implications for teachers’ professional development.

Experienced teachers broadly refer to those with years of teaching behind them and scholars suggested teachers with at least 4 to 5 years of teaching might be considered experienced teachers (Richards et al., 1998; Gatbonton, 1999). They lead the orientation of teaching reform and professional development (Yan and Wang, 2022). Despite rich experiences in teaching, many of them are digital immigrants (Prensky, 2001) who are not familiar with technology affordances and thus, are either reluctant or slow to use technology in teaching (Li, 2014; Huang et al., 2019; Huang F. et al., 2021). Many teachers need to strive to acquire familiarity with technology (Prensky, 2001). In the ERT context, what attitudes they hold, what identities they perceive, and how they coped with difficulties if any, is under-researched in the literature, to our best knowledge.

Based on the discussion above, this study aims to understand experienced university English teachers’ emotional attitudes toward ERT, unpack strategies they have adopted to cope with challenges, and their professional identities when conducting ERT. Specifically, the research questions are as follows.

1. What were university experienced English teachers’ emotional attitudes toward ERT?
2. How did they cope with challenges when teaching with technologies in the ERT context?
3. How did they perceive their professional identities in the ERT context?

In doing so, the study enriches the technology-assisted language teaching and learning literature by contextualizing the study in the emergency remote teaching context and provides suggestions for front-line teachers to achieve continuing professional development, especially in improving their technological, pedagogical, and content knowledge and skills, and coping with difficulties when using technologies.

## 2. Literature review

### 2.1. Language teachers’ perceptions of technology-assisted language teaching

The effectiveness of technologies in language teaching and learning has been well-recognized among English teachers (Dong and Ishige, 2022). For example, it transforms pedagogy, breaks the limitation of time and space, provides authentic language teaching and learning materials, facilitates communication, reduces students’ anxiety, and enhances their learning motivation (Yang and Chen, 2007; Shadiev and Yang, 2020; Huang F. et al., 2021).

In recent years, mobile devices (e.g., smartphones, laptops, and pads) have been increasingly adopted by university language learners. Due to the portability and affordance of mobile devices, mobile-assisted language learning (MALL) has become ubiquitous among university students who engage in learning not only in formal contexts but also in informal learning contexts, such as self-directed language learning beyond the class (Lai and Gu, 2011; Foomani and Hedayati, 2016; Gabarre et al., 2016; Taj et al., 2016). Research indicated that students are favorable to the use of mobile devices for language learning, and researchers have endeavored to study the effectiveness of mobile-assisted language learning (e.g., Lin and Lin, 2019). For example, students found the use of APP-learning flashcards to be a very efficient, convenient, and enjoyable vocabulary learning method (Davie and Hilber, 2015). A recent review study (Chen et al., 2020) suggested MALL is more effective than conventional methods and a medium-to-high effect size was indicated for the overall effectiveness of MALL. Other studies indicated that learner engagement is a chronic challenge to MALL adoption and is negatively impacted by small screen sizes and limited battery power and the life of mobile devices (Duman et al., 2015; Dashtestani, 2016).

Despite the popularity among students and effectiveness of MALL, studies suggested some English teachers feel reluctant to use technology including mobile devices in teaching (Batane and Ngwako, 2017; Teo et al., 2018) or used them at a low level (e.g., delivery of contents) instead of using mobile devices to transform pedagogy and maximize personalized teaching (Lai and Zheng, 2019). This is especially truer for experienced English teachers who have accustomed to traditional teaching and have accumulated sufficient knowledge and experience to teach without using technology. Different from novice and younger teachers who are eager to learn innovative teaching methods (i.e., technology integration in teaching), experienced EFL teachers were slow, unwilling to, and even rejective to change their teaching habits (Huang et al., 2019). In a long run, their technology adoption is still unsatisfying.

Scholars have been endeavoring to understand factors that influence teachers’ technology-using intention. For example, Ertmer (1999) and Ertmer et al. (2012) suggested teachers’ technology integration was influenced by internal and external factors. External factors such as equipment access, training, resources, and support were suggested as the first-order barriers; while internal factors such as confidence, beliefs about teaching and learning, beliefs about technology use, and willingness to change were considered as the second-order barriers (Ertmer, 1999). Later, Tsai and Chai (2012) suggested teachers’ technology uptake was also influenced by the third-order barrier, namely, lack of design thinking. Hsu (2016) examined the interconnections among the barriers and suggested the significant influence of TPACK (technological, pedagogical and content knowledge) on the perceived ease of use and perceived usefulness of mobile devices in language learning. Other studies contextualized in the EFL setting suggested EFL teachers perceived technology-related policies (Huang and Teo, 2020; Yan and Wang, 2022), cultural values (Ertmer and Ottenbreit-Leftwich, 2010; Huang et al., 2019), and constructivist language teaching beliefs (Teo et al., 2018) were significant factors that influenced English teachers’ intentions to use technology. Lai and Jin (2021) suggested teacher identity influenced their technology adoption and approaches to using technology.

### 2.2. Emergency remote teaching

Emergency remote teaching (ERT) refers to remote online teaching adopted by teachers to maintain teaching and learning in response to unexpected events or emergencies that break up normal school teaching (Hodges et al., 2020; Mohmmed et al., 2020). Different from traditional asynchronous online teaching where teachers and students do not have to be online at the same time but log in at different times and from different places (Songkram, 2017), ERT is a synchronous teaching mode that teachers and students meet, mostly in the videoconference environment. In such environments, teachers need to be competent in using diverse tools (e.g., mobile phones, pads, PC) to interact with students in real-time. Researchers suggested it is an emergent but so far understudied form of online education (Grammens et al., 2022).

In this study, the emergency refers to the COVID-19 pandemic that stopped formal teaching and learning. Teachers shifted from classroom-based teaching to pure online teaching supported by the Internet by using diverse tools such as laptops, smartphones, and personal computers. Different from traditional technology-enhanced teaching contexts where teachers are physically in the classroom and operate technology to assist teaching, ERT requires teachers to teach in the virtual class, deliver teaching and interact with students synchronously.

It’s reported that more than 1.5 billion university students in about 188 countries have shifted to ERT since the breakout of COVID-19 (Dong and Ishige, 2022). Recently some countries issued policies to implement formal school teaching and universities took measures to help students to return to in-class learning but, in many countries, especially developing countries with large population densities (e.g., China), governments and universities are still taking measures to prevent the potential large-scale infection, and thus, university teachers are conducting classroom-based teaching and ERT alternatively.

To sustain teaching and learning, a variety of online platforms have been designed. For example, EFL teachers in Chinese universities used videoconferencing tools such as *Tencent Meeting* and *Ding Talk* (Huang F. et al., 2021; Yan and Wang, 2022), which can be installed on mobile devices (laptops, smartphones, pads) and PC that enable teachers to meet students and colleagues online, deliver teaching, interact with students synchronously, provide instant feedback, and so on. Besides the above-mentioned tools, some teachers used *the Rain Classroom* to complete their teaching tasks. The features of traditional online teaching and the ERT were shown in Table 1.

**Table 1:** Features of online teaching and emergency remote teaching.

	Online teaching	Emergency remote teaching
Definition	The process by which a learner interacts with content or people *via* the Internet for learning (Means et al., 2014; Xie and Rice, 2021)	A distance online educational mode to cope with educational disruption caused by unexpected events (Hodges et al., 2020)
Purpose	To enhance learning and supplement face-to-face teaching (Gilbert, 2015).	To provide reliable, temporary, and fast access in crisis (Kirkwood and Price, 2014; Hodges et al., 2020; Mohmmed et al., 2020)
Delivery mode	Primarily asynchronous, few synchronous (Huang, 2018; Manfuso, 2020).	Primarily synchronous, few asynchronous (Manfuso, 2020)
Design process	Careful design with time investment (Hodges et al., 2020)	Unprepared and hasty processes including course design and device tests (Hodges et al., 2020; Yan and Wang, 2022).
Technical/Resource support	Well-designed ecosystem for learner engagement, and teacher/technical support (Manfuso, 2020)	Insufficient/not guaranteed timely technical support and resources (Mohmmed et al., 2020)

Although studies suggested that university English teachers generally hold positive attitudes toward technology use (Teo et al., 2018; Huang et al., 2019), these studies were conducted in contexts where teachers have the volition to use technology; in addition, participants diversify in their ages and research focus were not only on experienced teachers and thus findings of previous studies may be different from those in the ERT context. Based on the above, unpacking experienced EFL teachers’ emotional attitudes, strategies to conquer difficulties, and their professional identities are significant in both teacher education literature and technology-assisted language learning literature.

### 2.3. Emotional attitude, coping strategies and professional identity in ERT

Emotional attitude refers to one’s perception of an object, generally either positive or negative (Ajzen, 1991; Rauf and Suwanto, 2020). Several studies implied it is one of the most important constructs of technology integration (e.g., Kim et al., 2013; Teo et al., 2018). Teachers’ beliefs and attitudes were influenced by perceived usefulness, ease of use, and other factors such as innovativeness, technology complexity, and perceived anxiety (Venkatesh and Bala, 2008). Studies (Teo et al., 2016; Semerci and Aydin, 2018) indicated that the success of technology integration in the classroom heavily depends on teachers’ positive attitudes toward technology use. Given that educational technology is adopted as an alternative tool to assist Face-to-Face teaching, most teachers held positive attitudes toward technology use in class teaching before ERT (e.g., Huang et al., 2019). However, in ERT, technology was adopted as the only effective way for teachers to sustain teaching from disruption caused by unexpected events (Huang F. et al., 2021; Kim et al., 2022). The technology integration has escalated to a higher level, which put forward a new requirement for teachers’ technological, pedagogical, and content Knowledge (TPACK). Previous studies show that English teachers’ attitudes toward technology use in teaching change in terms of their gender, academic major, years of teaching with technology, and TPACK (Arcueno et al., 2021; Huang et al., 2022). It’s also indicated that the digital native teachers who have been well-educated in using technology or accustomed to using technology (Prensky, 2001) show more positive attitudes than the others. However, most of the experienced teachers in Chinese universities are not born to be digital natives. Compared to those younger teachers, they may hold diverse emotional attitudes toward ERT (Tzifopoulos, 2020).

A coping strategy is a behavioral reaction to reduce stress in adversity (Holahan and Moos, 1987; Carver et al., 1989). In existing studies on stress and coping strategies, scholars place foci on how effective coping strategies produce more positive outcomes and fewer negative ones (MacIntyre et al., 2020). If coping strategies did not work, individuals may find themselves producing negative emotions, such as stress and anxiety. Stress-related emotions generally lead to increased maladaptive behaviors. For teachers, especially those who have experienced professional burnout (Bottiani et al., 2019), the extent to which coping strategies were used would influence their attitudes and behaviors in teaching. Compared with technology-savvy younger teachers, elderly teachers with years of teaching experience may lack technical knowledge and perceive more stress while adapting themselves to the ERT (Huang et al., 2019). Therefore, investigating how experienced teachers coped with challenges in ERT is especially significant. Previous studies investigated teacher’s perceptions of challenges and coping strategies in ERT (Klapproth et al., 2020; MacIntyre et al., 2020; Trust and Whalen, 2020; Arcueno et al., 2021; Ghanbari and Nowroozi, 2022), such as planning, active coping, venting, and seeking for help, which was summarized into emotion-focused and problem-focused strategies (Lazarus and Folkman, 1984). However, very few inquired into how elderly experienced teachers coped with difficulties in the ERT context.

A teacher’s professional identity refers to the meaning of “who one is” as a professional and it relates to a teacher’s self-knowledge in teaching (Richardson and Watt, 2018). With the pandemic being an extreme example of the disruption of education as normal, teachers’ professional identity, essentially teachers’ beliefs of their roles, is more than ever set in a changing context and also influences teachers’ expectations, design thinking, and behaviors (Trent, 2019; Suarez and McGrath, 2022). According to Lai and Jin (2021), teachers’ professional identity has been identified as three aspects in relating to teachers’ roles. The first aspect is teachers’ perceptions of their roles in achieving educational goals, including instructor orientation and educator orientation, with the former paying attention primarily to developing students’ knowledge and skills for qualification purposes, and the latter with a broader interest in students’ well-being and whole person development. The second aspect relates to teachers’ roles in terms of instruction methods (van Veen et al., 2001). Student-centered instruction or teacher-centered instruction are typical examples of this aspect (Lai and Jin, 2021). The third aspect is teachers’ perceptions of their roles concerning the professional knowledge base. Teachers may perceive themselves as either subject matter experts or pedagogical expert (Pennington and Richards, 2016). A subject matter orientation is often associated with teacher-centered instruction that primarily focuses on the transmission of knowledge. Teachers with didactic and pedagogical expert orientations are more learner-centered in their instructions, focusing on constructing positive learning environments to improve students’ interest, inspire and facilitate students’ engagement in learning (Beijaard et al., 2000). Previous studies suggested teachers who held student-centered instruction are more likely to use technology in innovative ways beyond information delivery (Becker, 2000; Huang et al., 2019) and comparatively, teachers holding teacher-centered beliefs tend to use technology in a more regulated and restricted manner to primarily serve the instruction purpose of facilitating skill acquisition (Martin and Vallance, 2008).

Despite policy requirements to conduct ERT to ensure the continuity of teaching and learning during the pandemic period, studies suggested English teachers had reluctance and anxiety about the ERT (Ozamiz-Etxebarria et al., 2021; Pressley et al., 2021). There exposed a severe lack of teacher training for conducting ERT (Trust and Whalen, 2020). Many teachers scrambled to figure out how to shift their pedagogy to the ERT (Trust and Whalen, 2020). In this process, their professional identities may be reconstructed because teachers’ professional identity development is a continuous learning process where behavior, creation of related meaning, and social context interact (Suarez and McGrath, 2022).

## 3. Materials and methods

### 3.1. Participants

Considering EFL teachers’ perceptions of emergency remote teaching (ERT) may change during the teaching by learning process, we have conducted semi-structured in-depth interviews among participants as interviews provide direct routes to the research data through interactions (Drever, 1995). Convenience and purposive sampling techniques are combined when inviting participants, given we intend to get specific and rich information from a particular population of interest (experienced university English teachers but with no prior knowledge of ERT), but meanwhile, it is challenging to access many participants during the quarantine. Therefore, we invited experienced university English teachers with whom we are familiarize and willing to participate in the study. In this study, participants are experienced EFL teachers from the Shandong and Henan provinces of China and they were chosen based on the following criteria. First, they have been working as EFL teachers for at least 10 years but have no prior knowledge or experience with the ERT. Secondly, they teach diverse subjects in the English domain, such as translation, English teaching pedagogy, and English speaking. We believe these teachers are good representatives that lead to the depth and richness of understanding (Brannen and Nilsen, 2011; Baker and Edwards, 2012), because they meet our criteria of sampling and after these interviews, we did not find further new and valuable insights and therefore, stopped interviewing other teachers (Ragin, 2014).

It’s worth noting that all the participants were born before 1980 and had years of teaching experience. Although they had technology-using experiences in classroom-based teaching, they suggested themselves as neither technology-savvy users nor digital natives (Prensky, 2001), and they had very limited knowledge and experiences regarding technology-assisted language teaching. They suggested their technology usages were mostly at a behavioral level and to be specific, they use tools such as PowerPoint slides and social media tools (e.g., *WeChat* chatting groups; *Tencent meetings*) to deliver content. Table 2 showed detailed information about the participants of the study.

**Table 2:** Demographic information of interviewees (*N* = 5).

Pseudonym	Gender	Years of teaching	Teaching subjects	Platforms/Tools	Locations (Provinces)	Interview types
A	Female	30	Translation	Ding Talk, Rain Classroom, Tencent Meeting	Shandong	Face-to-Face
B	Female	29	Translation	MOOCs; Ding Talk, Rain Classroom, WeChat	Henan	Phone call; WeChat
C	Female	20	Pedagogy	MOOCs, Ding Talk, Rain Classroom, Xuexitong	Henan	Phone call; WeChat
D	Female	27	Public speaking; Translation	Ding Talk, Rain Classroom, Tencent Meeting	Shandong	Face-to-Face
E	Male	16	Linguistics; Writing	Ding Talk; Rain Classroom; Tencent Meeting	Shandong	Face-to-Face

### 3.2. Instrument

An interview protocol was designed and used in the interview process. The interview protocol was designed based on the authors’ understanding of literature, including technology acceptance theories, teacher identity, coping strategies (e.g., Shen, 2009; Chen and Mensah, 2018; Huang et al., 2019; Noonan, 2019), and the context of the study. A pilot interview was initially conducted, and questions were revised where necessary to ensure participants could understand, which indicated the validity of the interview questions.

The interview protocol includes two sections. The first part enquires into the interviewee’s demographic information, such as years of teaching, teaching subjects, and the platforms, or tools they used in teaching. The second part includes a series of questions inquiring into interviewees’ perceptions of emergent remote teaching, difficulties they have encountered, and coping strategies they adopted. Sample questions are: (1) How did you feel when you are told to switch your teaching online? (2) Did you encounter difficulties? How did you deal with them? (3) How do you describe yourself as an English teacher during the pandemic? The interview protocol was shown in the Appendix.

### 3.3. Procedure

The interviews were conducted either face-to-face or by phone given it is not easy for teachers to do during the pandemic period. The interviews were initially conducted in the spring and autumn semesters of 2020 when ERT emerged. As ERT continued in China, we did follow-up interviews in the spring of 2021 to better understand teachers’ perceptions. During the interviews, questions were asked based on the interview protocol, and additional questions were asked were necessary to explore deep the understanding of EFL teachers’ perceptions of ERT. Before the interview, interviewees were informed of the research purposes, their rights and freedom to withdraw at any time, as well as the confidentiality of their personal information. Generally, each interview lasted about 20 min. All the interviews were completed within 1 week.

Participants agree with the interview recording for further analysis. During the interviews, the authors took elaborate and detailed notes to retain thick data (Spradley, 2016). The authors transcribed interview materials word by word and sent them to interviewees for their confirmation of the contents.

### 3.4. Data analysis

Considering the reflective analytical depth and focus of this study, a manual encoding approach was adopted with paper and colorful marks. And the coding occurs in 3 stages based on the Grounded Theory (Walker and Myrick, 2006).

**Figure 1:**
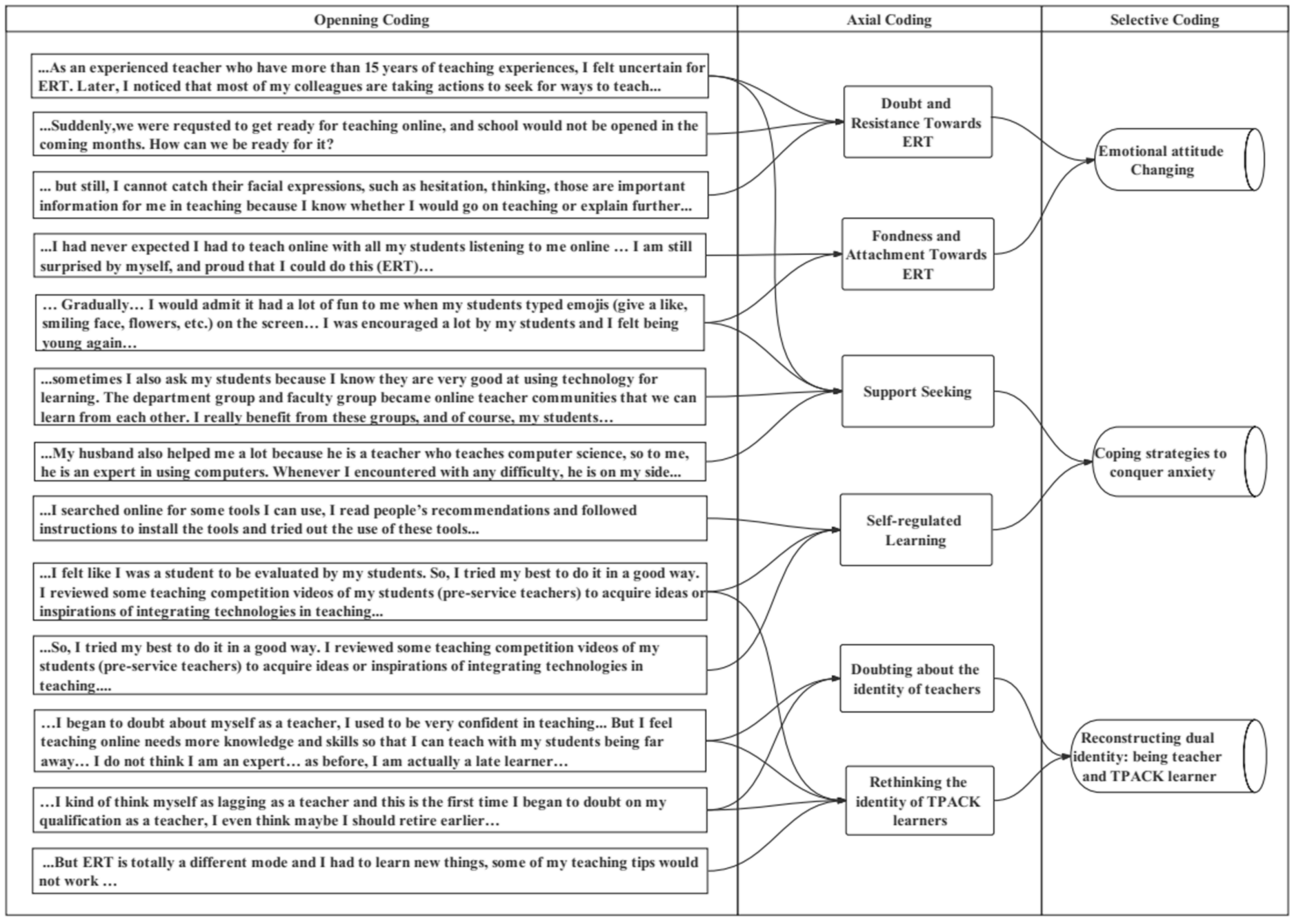
An example of the coding process.

The first open coding focused on informant-centric terms (Linneberg and Korsgaard, 2019) to avoid restriction by preconceived codes. The authors read the transcriptions and scrutinize the meanings of EFL teachers’ perceptions, and emerging codes inductively (Corbin and Strauss, 1990). The second and third stages (axial coding and selective coding) were more researcher-centric in which the research questions and related theories were taken into account. To be specific, the axial coding categorized, conceptualized, and lifted the open codes to a higher level of abstraction (e.g., concepts, dimensions, categories, themes; Gioia et al., 2013). In the selective coding, all the categories were connected to form core categories that ultimately represent the central themes of the research (Walker and Myrick, 2006). As Linneberg and Korsgaard (2019) suggested, the researcher must engage with the data continuously during the analysis, the immature themes or categories were generated, and we have repeatedly verified the accuracy of coding. Figure 1 illustrated the coding process.

To avoid bias and ensure the inter-rater reliability in the data analyses process (Miles and Huberman, 1994), the first author and second author transcribed the interviews word by word and sought verification from participants while coding transcripts; the fourth author checked the original statements; and then, the second and third author checked codes and themes from them. An agreement of about 90% in terms of the concepts and categories was reached in the coding process. Coding disagreements were discussed and reconciled through consultation between the authors to ensure inter-rater consistency, rigor, and credibility (Miles and Huberman, 1994).

## 4. Findings

### 4.1. Changing emotional attitudes: From doubt and resistance to fondness and attachment

The interviews suggested that experienced EFL teachers showed different attitudes to the ERT when they were initially informed to teach online during the COVID-19 outbreak. At that time, their attitudes were categorized as being positive, doubtful, and negative. However, for those who possessed negative attitudes (doubt, rejection) at the initial stage, their negative attitudes were gradually changed to the willingness and even fondness, especially when they realized ERT would not be *a flash-in-the-pan* phenomenon, as well as the effectiveness of technology use to sustain teaching in special situations.

Holding a positive attitude, teacher D realized the necessity of ERT in the pandemic and in her view, teachers should have positive attitudes toward the changing teaching context to ensure effective teaching and learning. She endeavored actively to cram herself with sufficient technical knowledge and skills as soon as being notified to teach online.

… *I learned how to use technology to teach while being informed of the necessity of ERT, including what apps or platforms I can resort to and how to use them. I think it is a good learning opportunity and actually, every teacher needs to get used to this, since teaching and learning cannot be suspended for a long time, but it was really a hurry learning, really challenging…* (From Teacher D)

However, some experienced teachers were unwilling to adopt ERT. Teacher C mentioned some teachers negotiated with school administrators about possibilities to postpone teaching till the school was open, by having make-up classes at weekends. Unfortunately, their requests were turned down and they had to obey rules.

Being ensured of the necessity of ERT, Teacher C had to take time to pick up technological skills and learn how to use them in teaching. She asked colleagues online from time to time when she encountered difficulties. To her surprise, she gradually realized the usefulness of using technology in teaching and her attitude toward ERT changed from resistance to openness and even fondness. She felt like a new door to teaching was open to her.

…*Suddenly, we were requested to get ready for teaching online, and school would not be opened in the coming months. How can we be ready for it? We did not receive such training…We, teachers, need to obey the policies because school leaders will check teachers’ ERT. So, I tried to learn, and sometimes I asked help from younger colleagues and even some of my students about the operation of some apps… It is interesting that, I did not expect I could pick up these skills at my age, although as a teacher who taught pedagogy, I know using technology is a trend and I taught my students of its importance because they are pre-service English teachers. But I had never expected I had to teach online with all my students listening to me online… I am still surprised by myself, and proud that I could do this (ERT)…* (From Teacher C)

Similar to Teacher C, Teacher B’s attitude toward ERT also showed a transition from uncertainty to enjoyment. Initially, she worried because she had formed a solid face-to-face teaching habit with years of on-site teaching, in addition, she lacked knowledge and experience in online teaching design and especially ways to motivate and organize students to interact with each other, which echoes previous studies that suggested design thinking is a critical factor that influenced teachers’ technology using attitudes and behavior (Tsai and Chai, 2012). Fortunately, she conquered uncertainty and difficulties and gained enjoyment and confidence. She also suggested the first-order barriers including facilitating conditions (Internet speed and stability) and support from skilled technology users (colleagues and students) and the second-order barriers (teaching beliefs, self-efficacy) were indispensable for her to succeed in ERT (Ertmer, 1999; Huang et al., 2019).

… *At the outset, we didn’t believe this teaching mode* (ERT) *would lead to a good effect, because we (teachers) have been accustomed to face-to-face teaching. How can I interact with my students? I know I can call their names, but still, I cannot catch their facial expressions, such as hesitation, and thinking, those are important information for me in teaching because I know whether I would go on teaching or explain further. I have been accustomed to face-to-face teaching, which is easy for teacher-student interaction. Think about ERT, students were far away from teachers, and you know, I would not turn on the camera all the time, so, how can we catch students’ reactions in time? … But since I have no other good ways to sustain teaching, I kind of force myself to attempt to learn new technologies* … *Gradually…I think I am doing pretty well, and I would admit it had a lot of fun for me when my students typed emojis (give a like, smiling face, flowers, etc.) on the screen… I was encouraged a lot by my students, and I felt young again…* (From Teacher B)

Despite the initial worries and concerns, most experienced teachers gradually acquired a fondness for ERT. But still, some teachers held a negative attitude toward ERT, and they were very slow to change. As Teacher E suggested to the authors, one of his colleagues disobeyed university policies and did not take on any teaching tasks during the pandemic.

*… Most of the teachers in my faculty tried their best to conduct ERT, but there were some exceptions. For example, one of my colleagues who taught literature did not believe in online teaching, so he did not take any classes during the period of suspension…* (From Teacher E)

### 4.2. Strategies to conquer anxiety and succeed in ERT

#### 4.2.1. Seek support from peers, students, and family

Lacking synchronous online teaching experience and training, English teachers felt the operation of ERT platforms and tools was complicated to learn. Initially, there was a high degree of perceived anxiety among experienced English teachers, but finally, they took action to succeed in ERT. During the process, teachers’ online cooperative community was gradually built up. And supports from their peers, technology-savvy students, and families played important roles for experienced teachers to conquer the anxieties and difficulties of ERT.

… *I felt so anxious because I had no online teaching experience, I did not even know what technologies or platforms I can use, not to say how to use…But you know at that time, every teacher had to learn, whether you like it or not, (sigh) those days. We (teachers) called each other, and comforted each other, … I called one of my colleagues who were close to me, she is younger and taught me how to use the Tencent meeting and Tencent classrooms, through WeChat. It really helped me a lot…* (From Teacher A)

At that moment, online teacher communities became the savior for teachers to sustain ERT. The young teachers or people who were familiar with technical operations actively shared their technical experience, and teacher A represented the experienced teachers in Face-to-Face teaching, however, lost their master role in the initial stage to marginalization. The following quotes elaborated on this perspective:

*…Our dean (who is good at technology) made a short video to share with us ways to use Ding Talk, a platform that can be used for teaching. Later, I knew there are many different tools and platforms, so I also asked my colleagues which is better, it saved my time, a shortcut, I would say…* (From Teacher A)

…Some teachers who are skilled in using technology helped others and at this special time, such curriculum groups or teams played a great role…we shared experiences and learned from each other… I teach writing and the most important thing for me is to figure out good ways to organize teaching activities online. So, we have discussed a lot about the choices of online writing platforms… (From Teacher D)

Faced with an uncertain situation, most teachers chose to huddle together for warmth to reduce emotional tension. Besides discussion within the teacher group (community), she also tried to seek help from his students who are young and born in the digital era. Because, in her perception, university students are very good at using technology for learning. Indeed, she received technical support from them. Taking students’ suggestions, teachers learned to use apps in the smart mobiles to check students’ attendance.

…My students taught me useful tools in smartphones or pads to conduct roll calls, you know, we (teachers) still need to check attendance. I learned how to check students’ attendance by using Ding Talk and Rain classroom… (From Teacher C)

*… During the winter holiday, news of online teaching was suddenly released and broke the Internet, we (teachers) had a discussion in the WeChat group about what to do… As an experienced teacher who has more than 15 years of teaching experience, I felt uncertain about ERT. Later, I noticed that most of my colleagues are taking action to seek ways to teach. We discussed a lot in different WeChat groups (faculty group, department group, and curriculum group), sometimes I also ask my students because I know they are very good at using technology for learning. The department group and faculty group became online teacher communities where we can learn from each other. I indeed benefit from these groups, and of course, my students…* (From Teacher E)

To maintain facilitated conditions, teacher B even paid for high-speed internet to avoid potential emergencies such as network disconnection. With a series of efforts, teacher B finally received much emotional support to hold ERT from students.

*… I ask my colleagues and students for help, and they are really helpful. I also pay for high-speed Internet just in case you know, technology or Internet broke… After several classes, I formed my confidence in using technologies, especially Ding Talk which allows playback so that my students and I can review content…I would admit it had a lot of fun when my students typed emojis (give a like, smiling face, flowers, etc.) on the screen and I also learned to use emojis to attract students… I was encouraged a lot by my students, and I felt young again…* (From Teacher B)

Besides support from peers and students, some teachers also got help from their family members. Taking Teachers C for example, her family members shared the same living and working space, which indeed helped her to seek help from family members. When she encountered technical trouble, her husband who had better technical skills helped her a lot. She received emotional and technical support from these significant others.

My husband also helped me a lot because he is a teacher who teaches computer science, so to me, he is an expert in using computers. Whenever I encountered any difficulty, he is on my side, which comforted me a lot… (From Teacher C)

#### 4.2.2. Self-regulated learning to sustain continuing professional development

Although significant others such as colleagues, students, and family members play important roles to help teachers to conquer technology anxiety and deal with difficulties, teachers suggested they have deeply realized, for the first time, the urgent need to renew knowledge and skills to survive teaching in the digital age. The ERT caused by unexpected events virtually forced teachers to renew teaching perceptions and approaches, which enhanced teachers’ self-regulated learning for continuing professional development (Timperley et al., 2008; de Vries et al., 2013).

Self-regulated learning is a learning process involving learner’s active participation in the aspects of meta-cognition, motivation, and learning behavior (Zimmerman, 2002). It involves self-motivation, task analysis, learning strategies, self-control, and reflection. Teacher E, for example, realized the necessity and complexity of ERT, as well as his lack of knowledge and skills to perform ERT, he decided to learn by himself to conquer difficulties.

*…It is better to rely on me instead of relying on others. After all, it is impossible for my colleagues to co-teach… It is a profound lesson for teachers like me, who previously show indifference to teacher training programs related to technology use. I searched online for some tools I can use, I read people’s recommendations and followed instructions to install the tools and tried out the use of these tools. When I encountered difficulties, I calmed down and searched online for solutions. Besides, I tried to search for existing online courses and evaluate the appropriateness for my course and my students. I realized I have dealt with many problems, and I actually learned a lot…* (From Teacher E)

*…I teach English pedagogy and there is a chapter about technology use in language teaching. My students practice and demonstrate technology-assisted teaching in my class, and I evaluate their teaching. You will never expect one day, I had to teach totally online. I felt like I was a student to be evaluated by my students. So, I tried my best to do it in a good way. I reviewed some teaching competition videos of my students (pre-service teachers) to acquire ideas or inspirations for integrating technologies into teaching. For example, I trained one student to participate in a teaching competition, and she won the first-class reword. In her teaching, she has used the interview technique to introduce students’ ideas about collecting things, which is very interesting. I invited her to introduce her teaching design to my current students. I realized my students liked this approach a lot…* (From Teacher C).

Teachers’ technology integration in language teaching was suggested as unsatisfying or being lower level in existing literature due to external and internal barriers (Ertmer et al., 2012; Tsai and Chai, 2012) as mentioned earlier. In ERT, the biggest challenge for language teachers is the effective ways to engage students to learn and interact with them. Teacher E tried to attract students by utilizing multimedia. i.e., playing pop songs at the beginning of the teaching. Similarly, Teacher B tried to know about students’ preferences and learned to interact with students by using an online learning community supported by mobile tools, such as WeChat group and QQ group.

*… WeChat and QQ groups on smartphones are very popular and useful. I have learned to discuss with my students and provide feedback to them even after class for translations that students did not understand…My students are willing to interact and perform better in the WeChat discussion group, they send messages to me and they type very fast…, I don’t think our communications were hindered by time or space, instead I became to realize we are closer…* (From Teacher B).

### 4.3. Reconstructing dual identity: Being a teacher and TPACK learner

Participants of this study were experienced English teachers who had formed their teaching beliefs and grasped solid content knowledge and effective skills to teach. Many were considered experts and role models in their universities. However, the ERT caused by the outbreak made them feel deficient in technology-related knowledge and skills, some even considered themselves as online-teaching marginal men (Jin, 2021).

*…I began to doubt myself as a teacher, I used to be very confident in teaching and I can teach well even without good preparation since all the contents are in my mind. But I feel teaching online needs more knowledge and skills so that I can teach with my students being far away… I do not think I am an expert… as before, I am actually a late learner…* (From Teacher A)

In classroom-based teaching, experienced English teachers perceive their main goals are to enhance student’s knowledge and skills, so as to improve students’ well-being and overall development. They recognized the importance of student-centered pedagogy (Teo et al., 2018) and sought for strategies to improve students’ engagement. Comparatively, in the ERT setting, English teachers admitted that due to insufficient self-efficacy and TPACK to teach online, it is good to achieve the bottom line, which was accomplishing teaching tasks smoothly. They endeavored to survive teaching online by either self-directed learning or resorting to technology-savvy peers, students, and even their family members to guarantee teaching by using online teaching tools.

*…I kind of think of myself as lagging as a teacher and this is the first time, I began to doubt my qualification as a teacher, I even think maybe I should retire earlier… As a teacher teaching pedagogy, I have trained many students to win the teaching competition, I am familiar with teaching assessment standards and I know what good teaching is, but I am not confident in conducting teaching online personally. I think I have to learn technical knowledge and obtain skills to teach, after all, I cannot always rely on others for help…* (From Teacher C)

*…Students were good at using technology, so I sometimes learn from them. We had to admit that times are changing, we (teachers) need to change and keep learning innovative teaching concepts and approaches or otherwise, we will be unqualified and in the long run, students may not choose the course I teach, and I might encounter with job transfer…* (From Teacher E)

*…Before COVID, we (teachers) are seen by students as content knowledge experts, I believe I can handle my teaching without any difficulties, I know how to deliver knowledge, arouse students’ interests, organize activities, etc. But ERT is totally a different mode and I had to learn new things, some of my teaching tips would not work …* (From Teacher A)

In the ERT process, experienced English teachers overcame anxieties and difficulties, and thought of the ERT as a learning opportunity. Some teachers recognized they should take back prejudice against online teaching and even took delight in teaching design and online interaction with students. They suggested they found “another side” of their students, especially those who seldom talk in face-to-face teaching but are active in online courses. Therefore, the ERT provided teachers with new angles for their teaching reflection.

*Gradually, I found that some students who used to be silent or indifferent in face-to-face classes became quick answer providers online. Maybe virtual space provides introverted students with a new opportunity to show themselves…after teaching online, we (teachers) discussed our students and the possibilities to change teaching design based on students’ individual traits…* (From Teacher C)

## 5. Discussion

This study inquired into experienced English teachers’ perceptions of emergency remote teaching (ERT) in the Chinese university context. Findings from in-depth interviews suggested that experienced teachers hold diverse attitudes toward ERT and interestingly, their attitudes demonstrated a changing trend, namely, from doubt and resistance to fondness and emotional attachment. Besides, English teachers adopted diverse strategies to cope with difficulties to sustain teaching and learning. In the ERT process, their identities were reconstructed.

As Ertmer (1999) suggested, attitude indicates personal beliefs and is one of the second-order barriers that influence teachers’ technology use and integration. This is also in line with the technology acceptance model (TAM) that suggested a significant association between attitude and intention to use technology (Davis, 1989; Huang M. et al., 2021). In this study, teachers who held positive emotional attitudes were more willing to adapt themselves to the changing teaching context to ensure effective teaching and learning during the pandemic. They even crammed themselves with TPACK knowledge as soon as possible. Although some teachers initially held doubtful attitudes and were unwilling to adopt ERT, when they were informed of the indispensability of online teaching, they followed institutional policies and got over psychological difficulties (e.g., anxiety) to pick up technical skills in teaching. Such compliance which was also suggested in previous studies (e.g., Teo et al., 2018) not only demonstrated the high-power distance in the Chinese educational culture (Hofstede, 2011) but also indicated teachers’ professional commitment and sense of teacher identity (Pennington and Richards, 2016). In the ERT process, their initially negative attitudes (worries, anxiety, uncertainty) had changed to positive ones, such as fondness and emotional attachment toward technology use in teaching.

English teachers adopted various coping strategies to coordinate their stress (Griffith et al., 1999; Austin et al., 2005) and sustain teaching and learning during the pandemic. The ERT caused by the unexpected events made teachers realize the importance and necessity to renew teaching perceptions and approaches, which further benefited their continuing professional development (Timperley et al., 2008; de Vries et al., 2013). They have adopted self-regulated learning strategies to cram knowledge (e.g., TPACK) and in this process, support from significant others plays a vital role in their success in ERT. To be specific, teachers sought for help from technology-savvy students, peers, and their families to deal with technical difficulties, and decrease psychological anxiety and stress. These indicated teachers adopted both emotion-oriented strategies (Klapproth et al., 2020; MacIntyre et al., 2020; Arcueno et al., 2021) and problem-oriented strategies (Lazarus and Folkman, 1984). Lacking technical knowledge and operational skills, experienced teachers lost their master roles in teacher groups at the initial stage of ERT. Teachers considered themselves as being deficient, especially in technology-related knowledge and skills, and become online teaching marginal persons (Jin, 2021). These findings echo previous studies (e.g., Huang et al., 2022) that suggested English teachers perceived themselves as competent in the content and pedagogical knowledge, but incompetent in technology-related knowledge and skills, especially the technological, pedagogical, and content knowledge (TPACK). Fortunately, teachers became TPACK learners and managed to conduct ERT by adopting effective coping strategies.

ERT broke the comfort zones of these traditionally so-called experienced teachers and brought about changes in their attitudes toward technology integration. Their teacher identity had also been reconstructed in this changing process. In line with Kozhabayeva and Boivin (2021) suggesting teachers’ uncertainty about online teaching and a de-professionalization of teacher identity, findings in this study suggested experienced university English teachers’ perceptions of expertise were weakened amid ERT, but ERT made them a pressing TPACK learner. They finally managed to pick up technological skills to sustain English teaching.

Consistent with, ERT deepened English teachers’ understanding of integrating technology in English teaching, especially the mixed usage of different tools to perform teaching. Experiences of non-volitional technology adoption made English teachers get rid of their technology-marginal image and gradually became technology- savvy users. This transition will lead to teachers’ passion for using innovative mobile tools in formal and informal settings to enhance students’ learning.

**Figure 2:**
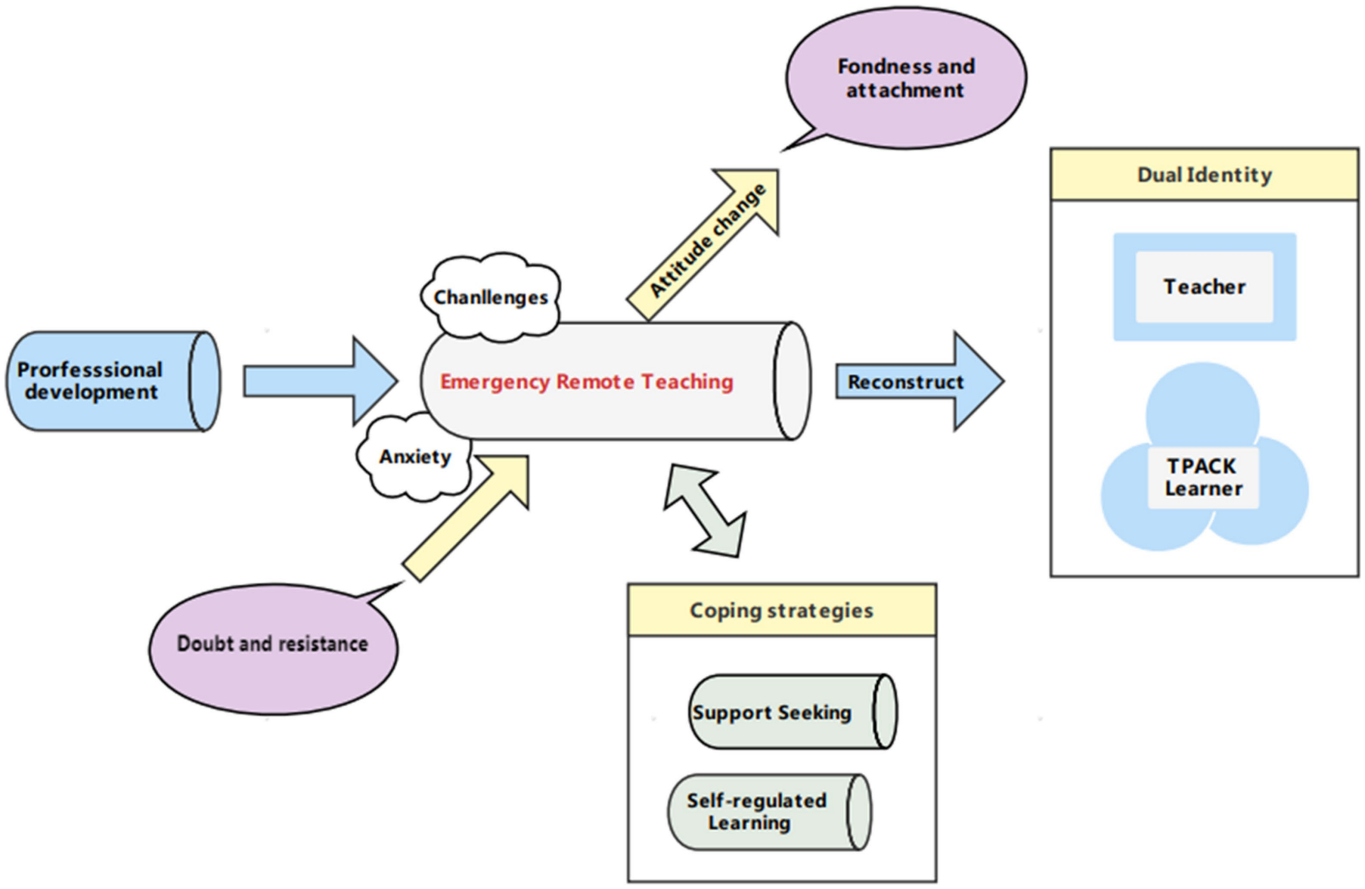
A systematization of teachers’ perception in ERT.

Overall, a systematization of teachers’ perceptions of ERT was illustrated in Figure 2.

## 6. Conclusion

This study examined experienced English teachers’ emotional attitudes toward ERT, their strategies to cope with difficulties, as well as the reconstructed dual identity during the process. As experienced and elderly university teachers, they were previously widely respected among students and colleagues for their expertise and authority in teaching English. ERT changed their self-cognition and de-professionalized teacher identity (Kozhabayeva and Boivin, 2021), and thus, they took measures to cope with difficulties. Results suggested although previously they had diverse attitudes toward technology-assisted teaching, their attitudes generally changed from reluctance to fondness. Experienced English teachers adopted diverse strategies to conquer perceived anxiety and difficulties (Venkatesh and Bala, 2008), during which, they reconstructed their teacher identities.

### 6.1. Implications

This study enriched existing literature on ERT and mobile-assisted language teaching and learning, and deepened people’s understanding of teachers’ perceptions of ERT by contextualizing the study in the Chinese university English teaching context.

The findings of the study provide empirical evidence for policymakers and teacher training program designers to make informed decisions to enhance teachers’ continuing professional development. For example, policymakers should take measures to provide timely technical support and design technology-oriented teacher-training workshops (e.g., mobile tools in teaching English) to improve teachers’ technological knowledge and skills, reduce technology anxieties, and further improve online teaching quality. Institutions can provide channels for university teachers to tell their worries, concerns, and difficulties and provide timely assistance to meet teachers’ emotional and psychological needs, reduce their anxiety and stress, and improve teachers’ well-being, Online teaching and learning communities should be paid more attention to as it was mentioned as one of the effective ways or platforms where teachers share ideas, seek help, and solve problems. ERT makes university experienced English teachers realize the importance to develop their professional skills through self-regulated learning. Since blended learning has become a new norm, experienced teachers are suggested to take initiatives to learn from those who have expertise in using technology, share learning experiences, reflect on their own teaching. This way, they will innovate pedagogy, pick up new skills, and adapt themselves to the changing teaching environment. They need to make the most of the features of technology tools and take on different roles to provide effective instruction in synchronous online teaching. In addition, teachers are suggested to consider students’ needs and favor of mobile tools in English learning and adopt mobile tools to design tasks so as to motivate their learning engagement and interactions.

### 6.2. Limitations and suggestions for further study

Considering the geographical diversity in China, results from the small and focused group may, to some degree, limit the generalization of results (Huang et al., 2019). The findings generated in this study may not be inclusive enough. Therefore, further studies were suggested to expand the number of interviewees to gain more comprehensive and deeper insights into experienced teachers’ perceptions of ERT.

## Data availability statement

The raw data supporting the conclusions of this article will be made available by the authors, without undue reservation.

## Ethics statement

The studies involving human participants were reviewed and approved by the School of Foreign Languages at the Qingdao University. The patients/participants provided their written informed consent to participate in this study.

## Author contributions

All authors listed have made a substantial, direct, and intellectual contribution to the work and approved it for publication.

## Funding

This study was supported by the Foreign Language Teaching and Research Council, China Association of Higher Education (Grant No: 21WYJYZD10) and the Ministry of Education under the National Emerging Humanities and Social Sciences Research and Reform Practice Project titled “Research on the educational model of students’ innovation ability” (Project No.: 2021110061).

## Conflict of interest

The authors declare that the research was conducted in the absence of any commercial or financial relationships that could be construed as a potential conflict of interest.

## Publisher’s note

All claims expressed in this article are solely those of the authors and do not necessarily represent those of their affiliated organizations, or those of the publisher, the editors and the reviewers. Any product that may be evaluated in this article, or claim that may be made by its manufacturer, is not guaranteed or endorsed by the publisher.

## Appendix

Interview protocol

- Basic information:
  - Would you please tell me about your teaching experience?
  - What course do you teach during the pandemic? What platforms did you adopt?
  - Do you have experience in using technology in teaching?
  - Have you received any training to conduct ERT?
- Questions about teachers’ perceptions of ERT.
  - How did you feel when you were told to teach online?
  - How did you prepare for your online teaching?
  - Did you encounter any difficulties? How did you deal with them?
  - How do you evaluate your online teaching? How is ERT different from traditional FtF teaching?
  - Would you please share with me your experience with ERT, for example, when using platforms or apps? How did you organize activities in ERT? How did you interact with students? Is it satisfying? Why? How do you describe the class atmosphere?
  - Have you ever communicated with or cooperated with your colleagues? If yes, when and how? Did you receive any support from your family, students, leaders, or school administrators? Would you please share your experiences and feelings?
  - Did you reflect your teaching and teachers’ identities when conducting ERT? How do you describe yourself as a teacher during the pandemic? Did you find any changes about teachers’ identities or roles?
